# Safety and Effect of the Use of Hydrotherapy during Labour: A Retrospective Observational Study

**DOI:** 10.3390/jcm12175617

**Published:** 2023-08-28

**Authors:** Elena Mellado-García, Lourdes Díaz-Rodríguez, Jonathan Cortés-Martín, Juan Carlos Sánchez-García, Beatriz Piqueras-Sola, Raquel Rodríguez-Blanque

**Affiliations:** 1 Research Group CTS-1068, Andalusia Research Plan, Junta de Andalucía, 18014 Granada, Spain; emg2684@gmail.com (E.M.-G.); cldiaz@ugr.es (L.D.-R.); jcortesmartin@ugr.es (J.C.-M.); beapiquerassola@gmail.com (B.P.-S.); rarobladoc@ugr.es (R.R.-B.); 2 Costa del Sol Health District, 29640 Fuengirola, Spain; 3 Department of Nursing, Faculty of Health Sciences, University of Granada, 18016 Granada, Spain; 4 Virgen de las Nieves University Hospital, 18014 Granada, Spain; 5 San Cecilio University Hospital, 18016 Granada, Spain

**Keywords:** hydrotherapy, labour, delivery, analgesia, pregnancy, maternal-foetal safety

## Abstract

Background: Hydrotherapy is a technique used for pain management during labour, but its safety for both the mother and foetus remains uncertain. Objective: The main aim of this study is to determine whether the use of hydrotherapy in the first stage of labour is safe for both the mother and newborn. Methods: A retrospective observational study was conducted to collect data from the partogram, maternal and neonatal history. Results: A total of 377 women who gave birth at the Costa del Sol Hospital in Malaga between January 2010 and December 2020 were randomly selected. They were divided into a control group (253 women) and an intervention group (124 women) that used hydrotherapy in the first stage of labour. There were no significant differences between the groups in terms of age, history of previous miscarriages, type of delivery, or newborn weight. The results showed that most women who opted for hydrotherapy were nulliparous, and the use of hydrotherapy during labour was safe for both the mother and foetus. There were no significant differences in the variables of maternal arterial hypotension, postpartum haemorrhage, postpartum maternal fever, foetal complications, neonatal admission, 1 and 5 min Apgar scores, umbilical arterial or venous pH, or foetal cardiotocographic recording. However, there was a significant difference (*p* = 0.005) in the rate of breastfeeding among mothers who opted for hydrotherapy (96% vs. 85.7%). Conclusions: The use of hydrotherapy during the first stage of labour is safe and is associated with increased breastfeeding rates compared to conventional delivery.

## 1. Introduction

The use of water as a therapeutic medium is not new, although its exact origins are not known. However, there is evidence of ancient cultures such as China, Egypt, Japan, Greece, and Rome using water as a treatment for physical and psychological ailments. In addition, there are bibliographic references [1] to clinical care that mention how, throughout history, immersion in water has been used during childbirth as a means of relaxation and pain relief.

Hydrotherapy is a method that focuses on pregnant women, especially during childbirth, which is why many women find it comfortable and useful [2]. In fact, the possibility of a water birth is a highly desirable experience for many families [3]. Women’s demands and the extremes to which some medical procedures can escalate have led to the search for alternatives for a natural and physiological birth. Water birth appears to empower and improve the skills of women who choose this option, and the use of hydrotherapy can enhance their experience during childbirth [4].

The benefits of water, such as buoyancy, hydrostatic pressure, and temperature, are relevant and beneficial to women during the birth process, as numerous studies have shown. These benefits include positive effects on the maternal experience of labour, such as reduced use of epidurals, improved ability to cope with pain, shorter labour, greater sense of control during the process, and increased comfort and ability to adopt comfortable positions [5]. A Cochrane review of 15 trials involving 3663 women, all comparing immersion with non-immersion, identified a number of physical and emotional benefits for labouring women associated with warm water immersion [1]. These benefits included a higher pain threshold, shorter labour, less medical intervention, greater relaxation, and overall satisfaction with the birth experience [1].

However, there is controversy about neonatal outcomes and baby safety. Associations such as the American College of Obstetricians and Gynecologists (ACOG) and the American Academy of Pediatrics (AAP) argue that the potential risks to the newborn outweigh the benefits for the woman.

The American College of Nurse-Midwives (ACNM) position statement on hydrotherapy in labour and birth recommends that women be provided with evidence-based information about water birth and that it be available to those with uncomplicated pregnancies who desire this option [5].

In response to the positions of the associations, current studies present data analyses that report on various neonatal clinical outcomes when comparing births without the use of hydrotherapy and births with the use of hydrotherapy. These studies do not suggest that outcomes are worse for babies born by water birth [6].

These findings are echoed in studies such as that by Davies et al. (2015), which concludes that water birth does not appear to be associated with adverse neonatal outcomes in a sample of low-risk women [6].

Another study by Bailey et al. (2020) concludes that water birth is not associated with an increased neonatal risk, nor is there a higher incidence of extensive perineal lacerations or postpartum haemorrhage [5].

Other specific adverse neonatal outcomes, such as respiratory distress, anaemia, sepsis, hypoxic-ischemic encephalopathy, asphyxia, or death, did not show significant differences between the groups that used water immersion and those that did not [7].

The Clinical Practice Guideline on Normal Childbirth Care in Spain, which is currently in force, recommends warm water immersion as an effective method of pain relief in the late stages of the first stage of labour [8]. This guide defines the different stages of labour: the latent phase (from the beginning of labour to 4 cm of cervical dilation), the active phase (from 4 to 10 cm of cervical dilation), the second stage of labour (expulsion), and the third stage of labour (placental delivery). It is important to take these stages into account when assessing the obstetric, maternal, and neonatal risks and benefits associated with the use of hydrotherapy during childbirth.

The hypothesis of this study is that the use of hydrotherapy during childbirth is safe for both mother and newborn and does not show significant differences in the variables analysed, including maternal arterial hypotension, postpartum haemorrhage, postpartum maternal fever, foetal complications, neonatal admissions, Apgar scores at 1 and 5 min, arterial or venous pH of the umbilical cord, and foetal cardiotocographic record. In addition, it is hypothesised that the use of hydrotherapy during childbirth may increase breastfeeding rates compared to conventional childbirth.

The main aim of this study was to determine whether the use of hydrotherapy in the first stage of labour is safe for both the mother and newborn.

Secondary objectives are:

- To study maternal perinatal outcomes.
- To study foetal outcomes of the use of hydrotherapy during labour.

## 2. Materials and Methods

### 2.1. Study Design

This is a retrospective observational study. The study was conducted in accordance with the Declaration of Helsinki for research on human subjects and was approved by the Ethics Committee of Costa del Sol Hospital (002_oct18_PI-hydrotherapy labour) in November 2018.

### 2.2. Setting

Women who gave birth at Costa del Sol Hospital between January 2010 and December 2020 were randomly selected from an anonymous database and retrospectively included in our observational study. The randomisation of the database was performed by the hospital’s IT department using computer software.

Data collection took place between September 2021 and May 2022 in the hospital’s clinical documentation unit, with data collected from the partogram, including clinical data on the mother, newborn, and delivery. For this purpose, a password-protected database was designed with various logical mechanisms to prevent the introduction of erroneous data.

Data analysis was performed from June 2022 to September 2022.

### 2.3. Participants

The inclusion criteria for the study were low-risk pregnant women, at term, with a singleton pregnancy, cephalic presentation, and need for hospital admission for delivery, with induction or spontaneous onset of labour.

The study was carried out on patients who gave birth at the Costa del Sol Hospital in Málaga.

Two groups were formed: the intervention group, consisting of women who used hydrotherapy during the first stage of labour, and a second control group who did not use hydrotherapy during labour.

Exclusion criteria were pregnant women with a twin pregnancy, pregnancies that began prematurely (<37 weeks), or post-term (>42 weeks).

### 2.4. Variables

#### 2.4.1. Sociodemographic and Anthropometric Variables

Age: the age of participants was recorded in years.

Parity: The GAPV formula (pregnancy-miscarriage-live birth) was used to record the parity of the pregnant women who participated in the study. For data analysis, it was classified as Nulliparous (no previous births) and Multiparous (one or more previous births).

#### 2.4.2. Variables Related to the Intervention

Type of delivery: the categories considered for the end of the second stage of labour were spontaneous vaginal delivery, vacuum-assisted delivery, forceps-assisted delivery, and caesarean section.

The duration of the use of hydrotherapy during the first and/or second stage of labour was defined in minutes.

#### 2.4.3. Maternal Outcome Variables

Postpartum perineal: classified as intact, first-degree tear, second-degree tear, third-degree tear, fourth-degree tear [9], and episiotomy.

Maternal hypotension: recorded in millimetres of mercury (<90/60 mmHg).

Fever: for analysis, fever was recorded if the temperature was >38 °C; no fever if the temperature was <38 °C.

Postpartum haemorrhage: yes/no.

Breastfeeding: yes/no.

#### 2.4.4. Variables Related to Foetal and Neonatal Outcomes

Foetal cardiotocographic recording during dilation and expulsion, data collected according to the clinical practice guidelines of the National Health System [8]: reassuring CTG, non-reassuring CTG, abnormal CTG.

Neonatal weight: measured in grams.

Apgar score [10] at 1 and 5 min after birth.

Venous and arterial pH.

Admission to neonatal unit: yes/no.

### 2.5. Bias

In order to avoid bias in the collection of data related to the variables to be considered, a database (Db) was designed, protected by a key, and equipped with various logical mechanisms to prevent the introduction of false data. Only the researchers involved in this project could access the database. It should be noted that the identifying data of the patients were separated in a second Db with a different access key. In this latter Db, only the principal investigator had access, and all researchers were committed to respecting the confidentiality of the data in accordance with the Personal Data Protection Law of 8 November 2018, and Law 41/2002 of 14 November, which is fundamental and regulates the autonomy of patients and their rights and obligations in terms of clinical information and documentation.

### 2.6. Study Size

The sample size calculated for this study was 248 pregnant women, with 124 in the control group and 124 in the hydrotherapy group (assuming a loss rate of 10% in the medical history assessment, with 80% power). The sample was later increased to 377 women as it was considered necessary to subdivide the main groups in order to extend and improve the study.

### 2.7. Statistical Methods

Descriptive analysis was performed using measures of central tendency, dispersion, and position (median and interquartile range (P75–P25)) for quantitative variables and frequency distribution for qualitative variables. To assess differences between study groups (absence vs. presence of hydrotherapy), the chi-squared test (or Fisher’s exact test if expected frequencies were less than 5) was used for qualitative variables, while Student’s *t*-test (or Mann–Whitney U test if the distribution was non-normal) was used for quantitative variables. Multivariate logistic regressions were performed between the following variables: postpartum haemorrhage, postpartum perineal status (comparing intact vs. first-degree tear, second-degree tear, third-degree tear, and episiotomy), foetal complications, neonatal admission, abnormal RCTGF signs, maternal arterial hypotension, and maternal fever. Each model was adjusted for age and number of pregnancies (1, 2 and 3 or more).

For all analyses, the level of statistical significance was set at *p* < 0.05. The analysis was performed using SPSS vs. 28.0 program for Windows (IBM Corporation, Armonk, NY, USA) statistical software.

### 2.8. Intervention

The intervention consisted of collecting data from patients who used hydrotherapy in the first stage of labour to compare with data of patients who did not use hydrotherapy.

The data were directly collected from the paper partogram in the pregnant woman’s medical record. The clinical documentation unit allowed personal access to the data. Once the data were obtained, statistical analysis was performed.

## 3. Results

After analysing the collected data in this study, we observed notable characteristics in the sample, particularly differences in previous pregnancies and childbirths of the women under analysis that showed statistically significant variations. These data are shown in Table 1.

On the other hand, a descriptive analysis was conducted regarding the time spent using hydrotherapy among women who chose this technique during the first stage and/or second stage of labour. The results are presented in Table 2.

### 3.1. Perinatal Complications

Among the maternal outcome data regarding postpartum haemorrhage, it was observed that 2.4% of pregnant women who did not receive hydrotherapy experienced postpartum haemorrhage, compared to 4.8% of women who received hydrotherapy. The *p*-value of the Fisher exact test is 0.22, indicating that the differences are not significant.

### 3.2. Safety of Hydrotherapy for the Mother

The presence or absence of intact perineal status was examined for differences between the groups (*p* = 0.651), and no statistically significant differences were found (Table 3).

It was investigated whether the use of hydrotherapy affected maternal hypotension. No significant differences were found between the groups (*p* = 1.000), suggesting that there is no significant relationship between maternal hypotension and the use of hydrotherapy. In the group that did not use hydrotherapy, 5.1% had maternal hypotension, whereas in the group that used hydrotherapy, 5.6% had maternal hypotension.

The presence of maternal fever in the postpartum period was analysed, and no statistically significant differences (*p* = 0.550) were found between the groups.

### 3.3. Foetal Safety of Maternal Use of Hydrotherapy

Analysis of neonatal outcomes showed no statistically significant differences between the groups in terms of neonatal complications or neonatal admissions (*p* = 0.540). There were no complications in 96% of newborns from mothers who chose to use water therapy during labour, and in 97.2% of cases where water therapy was not used. There were no differences in neonatal admissions (*p* = 0.846); however, the hydrotherapy group had a lower percentage of newborns admitted to the neonatal unit (4.8% vs. 5.9% in the non-hydrotherapy group).

In both groups, Apgar scores were analysed at 1 min and 5 min after birth. Apgar scores analysed at 1 min after birth showed no significant differences between the groups (*p* = 0.782). In the group that did not receive hydrotherapy, one newborn had an Apgar score of two, indicating severe neonatal depression at birth, and two newborns had Apgar scores between four and six, indicating moderate depression. The neonates in this group had normal Apgar scores between seven and ten in 98.8% of cases. In the hydrotherapy group, one newborn had an Apgar score between four and six, indicating moderate depression, and 99.2% of this group had scores between seven and ten, indicating normal newborn status.

In the analysis of the Apgar scores at 5 min in both groups, scores between seven and ten points were obtained, with the highest scores (ten points) observed in 98.4% of cases in the group that used hydrotherapy and in 96.4% of cases in the group that did not use hydrotherapy during delivery. No significant differences were found in the analysis, indicating good health of the newborns in the group that used hydrotherapy.

No statistically significant differences were found in the results of the foetal cardiotocographic recording (RCTGF) (*p* = 0.234). Calming RCTGF were present in 90.1% of the hydrotherapy group and 94.4% of the hydrotherapy group. In the group that did not use hydrotherapy, three RCTGF were abnormal and 22 were not sedative. These numbers decreased in the group that used hydrotherapy, where there were no abnormal RCTGF and seven non-sedating RCTGF.

The neonates’ venous and arterial pH levels were recorded. There were no statistically significant differences in venous pH between the groups (*p* = 0.490), with values of pH = 7.35 ± 0.071 in the group of women who received hydrotherapy and pH = 7.353 ± 0.064 in the group of women who received conventional treatment. The arterial pH values were also not significant (*p* = 0.400), with values of pH = 7.291 ± 0.086 in women in the hydrotherapy group compared to pH = 7.287 ± 0.088 in women who did not receive hydrotherapy.

Significant differences (*p* = 0.005) were found in favour of the hydrotherapy group who decided to breastfeed (96% vs. 85.7%). The comparison between the two groups showed that more women in the hydrotherapy group breastfed their newborns.

### 3.4. Multivariate Analysis to Reduce Risk of Bias

In the multivariate logistic regression model related to the presence of postpartum haemorrhage, hydrotherapy patients have an odds ratio (Exp(B)) of 2.487 (95%CI 0.75–8.22), although not significant (*p* = 0.135). The same is true for postpartum perineal status, which has an odds ratio (Exp(B)) of 1.039 (95%CI 0.64–1.68), which is not significant (*p* = 0.876). For the presence of foetal complications, these patients had an odds ratio (Exp(B)) of 1.533 (95%CI 0.46–5.10), not significant (*p* = 0.486). For increased neonatal attendance in the neonatal unit, hydrotherapy patients had an odds ratio (Exp(B)) of 0.796 (95%CI 0.29–2.16), not significant (*p* = 0.655). For the presence of foetal cardiotocographic record (FCRCF) in hydrotherapy patients, the odds ratio (Exp(B)) was 0.561 (95%CI 0.23–1.37), not significant (*p* = 0.486). The presence of maternal hypotension in hydrotherapy patients was also not significant (*p* = 0.655) and its odds ratio (Exp(B)) was 1.217 (95%CI 0.46–3.21). Finally, the presence of maternal fever in hydrotherapy patients was also not significant (*p* = 0.203) and its odds ratio (Exp(B)) was 2.940 (95%CI 0.177–49.01).

## 4. Discussion

In this study, we aimed to determine the safety of using hydrotherapy during childbirth for both the mother and newborn. We conducted a retrospective observational study at the Costa del Sol Hospital in Malaga between January 2010 and December 2020. During this period, 377 women were randomly assigned to either a control group or an intervention group that received hydrotherapy during childbirth. The results showed that hydrotherapy during childbirth was safe and did not present significant differences in maternal arterial hypotension, postpartum haemorrhage, postpartum maternal fever, foetal complications, newborn admissions, Apgar scores at 1 and 5 min, arterial or venous pH of the umbilical cord, or foetal cardiotocography. In addition, a significant increase was observed in the rate of breastfeeding among mothers who opted for hydrotherapy.

According to our results, although we did not find significant differences in terms of types of delivery, there was a lower incidence of dystocic deliveries in the group that used hydrotherapy. These findings are consistent with previous studies, such as that of Liu et al. [11], who also reported a significant decrease in the caesarean rate among the group that used hydrotherapy during childbirth. Similarly, Herrera et al. [12] found results similar to ours, where most deliveries ended spontaneously, and no significant differences were observed between the groups. Furthermore, a randomised controlled trial conducted by Cluett et al. [13] also reported that immersion in water had no significant effects on the rates of surgical delivery.

Regarding the parity of pregnant women, i.e., whether they are primiparous or multiparous, our results show that the intervention group had a high percentage of primiparous women (45%). However, this finding contrasts with other studies, such as Lewis et al. [14], who found significant differences among multiparous women, These women were more likely to use hydrotherapy and give birth in water. Despite this, both studies concur that hydrotherapy increases the chances of having a spontaneous vaginal delivery by respecting the natural progression of labour and minimizing unnecessary interventions. The difference between our results and those of Lewis et al. [14] may be due to a greater culture of hydrotherapy use among pregnant women, as it is a “young” technique in Spain. Cluett et al. [1] concluded that “water birth, under the supervision of a midwife, can be an option for slow labour progression, reducing the need for obstetric intervention.” They also reported that the use of hydrotherapy can reduce the rate of caesarean sections by providing personalised care to the woman during childbirth.

Our results include information on the effects that the use of hydrotherapy in childbirth can have on maternal health. These findings are supported by other studies in which no adverse effects were observed for the mother [1,11,12,15,16,17]. For example, Herrera et al. [12] did not observe infectious effects such as endometritis, puerperal fever, or puerperal sepsis. In our study, there were no significant differences in fever occurrences between the group that used hydrotherapy and the group that did not. Lim et al. [17] conducted a retrospective review in Singapore and found no significant differences in terms of postpartum haemorrhage, maternal infection, and perineal tears. They concluded that water birth does not seem to be associated with adverse maternal health outcomes. Gayiti et al. [18] conducted a retrospective study and did not find an increased risk for mothers in births where hydrotherapy was used.

According to the results of two meta-analyses, one conducted by Taliento et al. [19], including data from 212,843 women, and another conducted by Burns et al. [20], compiling the results of 15 studies with data from 63,891 women, both agree that the group that used hydrotherapy during labour had a lower risk of postpartum haemorrhage than the group that did not use it. Additionally, an observational cohort study [21] with a sample of 46,088 women also reported an association between water birth and a reduction in the incidence of postpartum haemorrhage. However, in our study, although we did not find significant differences, we observed a higher incidence of postpartum haemorrhage in the group that used hydrotherapy. We believe that this may be due to the active or physiological management of the third stage of labour. In our study, since no delivery occurred in water, the third stage was carried out outside of the water without the use of hydrotherapy, similar to the group that did not use it.

Furthermore, we found that the use of hydrotherapy is not related to a higher risk of tears, and there is also a lower number of episiotomies in women who use hydrotherapy. These results are supported by our study and other recent studies [1,3,20,22]. However, we also encountered a contrasting report by Bovbjerg et al. [23], who reported an 11% increase in the likelihood of experiencing some perineal trauma. They claim that this finding requires further investigation and could be a misclassification bias among women with complicated deliveries and the suspension of hydrotherapy use.

In this study, our results are consistent with previous research on the use of hydrotherapy in relation to maternal outcomes, such as perineal status and maternal infection. In 2014, the American Academy of Pediatrics (AAP) and The American College of Obstetricians and Gynecologists (ACOG) published a clinical report indicating the possible maternal benefits of hydrotherapy during the first stage of labour. However, there was controversy regarding neonatal outcomes in births with the use of hydrotherapy.

We can state that, with respect to neonatal outcomes and current scientific evidence, multiple comparative studies between births with hydrotherapy and traditional births have concluded and ensured that water birth is not associated with a higher incidence of adverse neonatal outcomes. Various types of studies from different sources [11,17,18,23,24] support similar morbidity and mortality results in groups that have used hydrotherapy compared to those who have not. An example is the Cochrane review [1], which found no evidence of increased adverse effects in births with the use of hydrotherapy.

In our study, we did not find significant differences in Apgar scores between newborns in both groups. This result is consistent with several previous studies [1,11,12,23,25,26] that also found no significant differences in Apgar scores and showed adequate extrauterine adaptation without infectious adverse effects. In addition, Mallen et al. [25] concluded that there is a lower risk of infection in the group that uses hydrotherapy. Other studies [1,11,15,16,23,27,28] also reported no significant differences in neonatal infection rates, suggesting that water birth does not carry a higher risk, and that there were no significant differences in arterial and venous pH results or in admissions to the neonatal intensive care unit [1,20,23,25,26,27].

Multivariate analysis was performed due to the large number of variables used in the study, to avoid confounding bias in this type of design. None of the models showed statistical significance for the presence of hydrotherapy. This is highly significant in our study as it indicates that the use of hydrotherapy during labour does not present a greater risk than labour without the use of hydrotherapy for experiencing any of the complications expressed in the study variables.

## 5. Conclusions

It is important to note that this study was conducted in a specific hospital and its results may not be generalisable to other settings. However, the findings are promising and suggest that hydrotherapy during labour can be a safe and effective option during labour and delivery. Additionally, the use of hydrotherapy during labour may also have additional benefits, such as increasing rates of breastfeeding.

In conclusion, the study suggests that the use of hydrotherapy during the first stage of labour is safe and may have additional benefits, such as increased breastfeeding rates.

In summary, research on hydrotherapy during labour is ongoing, and more studies are needed to fully understand its safety and effectiveness.

## Figures and Tables

**Table 1:** Comparison of Characteristics between Patients who Received Hydrotherapy and those who Did Not.

	Hydrotherapy				*p*
	Absence		Presence		
	*n*	%	*n*	%	
Total	253	67.1	124	32.9	
Age					
Mean—SD	32.5	5.3	31.7	5.6	0.103
Gestation					
1	81	56.6	62	43.4	0.002
2	94	71.2	38	28.8	
3 or more	78	76.5	24	23.5	
Miscarriages					
Absence	201	67.9	95	32.1	0.62
Presence	52	64.2	59	35.8	
Previous births					
0	94	55	77	45	<0.001
1	109	73.2	40	26.8	
2 or more	50	87.7	7	12.3	
Type of delivery					
Normal	236	93.3	121	97.6	0.132
Dystocic	17	6.7	3	2.4	
Newborn weight (grams)					
Mean—SD	3321.9	446.3	3306.3	378.8	0.369

**Table 2:** Time using hydrotherapy.

n	124
Mean	86.66
Median	62.5
Standard deviation	82.69
Minimum	10
Maximum	615
Percentile 25	45
Percentile 75	108.75

**Table 3:** Perineal Status No Hydrotherapy vs. Hydrotherapy.

Hydrotherapy	No Hydrotherapy	Perineal Status
39 (31.5%)	87 (34.4%)	Intact
45 (36.3%)	82 (32.4%)	1st degree
31 (25.0%)	57 (22.5%)	2nd degree
1 (0.8%)	3 (1.2%)	3rd degree
8 (6.5%)	24 (9.5%)	Episiotomy

## Data Availability

Data regarding the study are available upon request to the corresponding author.

## References

[1] Cluett E.R., Burns E., Cuthbert A. (2018). Immersion in water during labour and birth. Cochrane Database Syst. Rev..

[2] Hinkson L., Henrich W. (2018). Intrapartum care. J. Perinat. Med..

[3] Ulfsdottir H., Saltvedt S., Georgsson S. (2019). Women’s experiences of waterbirth compared with conventional uncomplicated births. Midwifery.

[4] Fair C.D., Crawford A., Houpt B., Latham V. (2020). After having a waterbirth, I feel like it’s the only way people should deliver babies: The decision making process of women who plan a waterbirth. Midwifery.

[5] Bailey J.M., Zielinski R.E., Emeis C.L., Kane Low L. (2020). A retrospective comparison of waterbirth outcomes in two United States hospital settings. Birth.

[6] Davies R., Davis D., Pearce M., Wong N. (2015). The effect of waterbirth on neonatal mortality and morbidity: A systematic review and meta-analysis. JBI Database Syst. Rev. Implement Rep..

[7] Milton R., Sanders J., Barlow C., Brocklehurst P., Cannings-John R., Channon S., Gale C., Holmes A., Hunter B., Paranjothy S. (2021). Establishing the safety of waterbirth for mothers and babies: A cohort study with nested qualitative component: The protocol for the POOL study. BMJ Open.

[8] Grupo de Trabajo de la Guía de Práctica Clínica Sobre Atención al Parto Normal (2010). Plan de Calidad para el Sistema Nacional de Salud del Ministerio de Sanidad y Política Social. Guía de Práctica Clínica de Atención al Parto Normal.

[9] Sociedad Española de Ginecología y Obstetricia (2020). Lesión obstétrica del esfínter anal. Otros desgarros perineales 2019. Prog. Obstet. Ginecol..

[10] Apgar V. (2015). A proposal for a new method of evaluation of the newborn infant. Anesth. Analg..

[11] Liu Y., Liu Y., Huang X., Du C., Peng J., Huang P., Zhang J. (2014). A comparison of maternal and neonatal outcomes between water immersion during labor and conventional labor and delivery. BMC Pregnancy Childbirth.

[12] Herrera A. (2021). La hidroterapia durante el parto. Cuidados de la Enfermería Obstétrica y sus resultados materno-infantiles. Conoc. Enferm..

[13] Cluett E.R., Pickering R.M., Getliffe K., Saunders N.J.S.G. (2004). Randomised controlled trial of labouring in water compared with standard of augmentation for management of dystocia in first stage of labour. BMJ.

[14] Lewis L., Hauck Y.L., Butt J., Hornbuckle J. (2018). Obstetric and neonatal outcomes for women intending to use immersion in water for labour and birth in Western Australia (2015–2016): A retrospective audit of clinical outcomes. Aust. New Zealand J. Obstet. Gynaecol..

[15] Mollamahmutoglu L., Moraloglu O., Ozyer S., Su F.A., Karayalcin R., Hancerlioglu N., Uzunlar O., Dilmen U. (2012). The effects of immersion in water on labor, birth and newborn and comparison with epidural analgesia and conventional vaginal delivery. J. Turk. Ger. Gynecol. Assoc..

[16] Zanetti-Dällenbach R., Lapaire O., Maertens A., Frei R., Holzgreve W., Hösli I. (2006). Water birth: Is the water an additional reservoir for group B streptococcus?. Arch. Gynecol. Obstet..

[17] Lim K.M.X., Tong P.S.Y., Chong Y.-S. (2016). A comparative study between the pioneer cohort of waterbirths and conventional vaginal deliveries in an obstetrician-led unit in Singapore. Taiwan J. Obstet. Gynecol..

[18] Gayiti M.R.Y., Li X.Y., Zulifeiya A.K., Huan Y., Zhao T.N. (2015). Comparison of the effects of water and traditional delivery on birthing women and newborns. Eur. Rev. Med. Pharmacol. Sci..

[19] Taliento C., Tormen M., Sabattini A., Scutiero G., Cappadona R., Pantaleo G. (2022). Impact of waterbirth on post-partum hemorrhage, genital trauma, retained placenta and shoulder dystocia: A systematic review and meta-analysis. Eur. J. Obstet. Gynecol. Reprod. Biol..

[20] Burns E., Feeley C., Hall P.J., Vanderlaan J. (2022). Systematic review and meta-analysis to examine intrapartum interventions, and maternal and neonatal outcomes following immersion in water during labour and waterbirth. BMJ Open.

[21] Aughey H., Jardine J., Moitt N., Fearon K., Hawdon J., Pasupathy D., Urganci I., Harris T., NMPA Project Team (2021). Correction to: Waterbirth: A national retrospective cohort study of factors associated with its use among women in England. BMC Pregnancy Childbirth.

[22] Haslinger C., Burkhardt T., Stoiber B., Zimmermann R., Schäffer L. (2015). Position at birth as an important factor for the occurrence of anal sphincter tears: A retrospective cohort study. J. Perinat. Med..

[23] Bovbjerg M.L., Cheyney M., Everson C. (2016). Maternal and Newborn Outcomes Following Waterbirth: The Midwives Alliance of North America Statistics Project, 2004 to 2009 Cohort. J. Midwifery Womens Health.

[24] Camargo J.C., Varela V., Ferreira F.M., Pougy L., Ochiai A.M., Santos M.E., Grande M.C.L. (2018). The Waterbirth Project: São Bernardo Hospital experience. Women Birth.

[25] Mallen-Perez L., Roé-Justiniano M.T., Colomé Ochoa N., Ferre Colomat A., Palacio M., Terré-Rull C. (2018). Uso de hidroterapia durante el parto: Evaluación del dolor, uso de analgesia y seguridad neonatal. Enferm. Clin..

[26] Peacock P.J., Zengeya S.T., Cochrane L., Sleath M. (2018). Neonatal Outcomes Following Delivery in Water: Evaluation of Safety in a District General Hospital. Cureus.

[27] Geissbuehler V., Stein S., Eberhard J. (2004). Waterbirths compared with landbirths: An observational study of nine years. J. Perinat. Med..

[28] Young K., Kruske S. (2013). How valid are the common concerns raised against water birth? A focused review of the literature. Women Birth.

